# Ultrasonographic assessment of thenar muscles for diagnosing sarcopenic obesity in patients with schizophrenia

**DOI:** 10.3389/fpsyt.2025.1698379

**Published:** 2025-12-04

**Authors:** Guoyu Yan, Huaying Ding, Xia Lin, Zhouyu Li, Lanlan Chen, Lan Xiang, Xiaoyan Chen, Youguo Tan

**Affiliations:** North Sichuan Medical College, Nanchong, China

**Keywords:** sarcopenic obesity, schizophrenia, ultrasonic diagnosis, adverse outcome, thenar muscles

## Abstract

**Background:**

Patients with schizophrenia have a high prevalence of sarcopenic obesity (SO) (19.2%), largely due to antipsychotic use and sedentary lifestyle. Conventional diagnostic techniques (e.g., dual-energy X-ray absorptiometry [DXA]) are limited by their cost and complexity.

**Objective:**

To evaluate thenar muscle ultrasound (thickness and echo intensity) combined with sex for SO screening and its association with pneumonia and falls.

**Methods:**

A total of 490 patients with stable schizophrenia underwent bilateral thenar ultrasonography. SO was diagnosed according to the Asian Working Group for Sarcopenia (AWGS) 2019 criteria (muscle mass, grip strength, gait speed) and the Japanese obesity consensus (body fat ≥20% in males, ≥30% in females, or visceral fat ≥100 cm²). Diagnostic efficacy (ROC analysis) and adverse outcomes were assessed.

**Results:**

Patients with SO (n=94) had significantly reduced thenar muscle thickness (left: 13.81 vs. 14.75 mm, p<0.001; right: 14.83 vs. 15.96 mm, p<0.001) and higher echo intensity (left: 39.57 vs. 35.66, p=0.002; right: 38.52 vs. 34.51, p<0.001) compared with patients without SO (n=396). A model combining right thenar thickness, echo intensity, and gender achieved the best diagnostic performance (AUC = 0.805, sensitivity=73.2%, specificity=76.3%). SO was significantly associated with increased fall risk (adjusted OR = 2.889, p=0.002) and pneumonia (unadjusted OR = 2.175, p=0.038).

**Conclusion:**

Thenar ultrasound combined with sex provides an efficient tool for SO screening (AUC>0.8) in patients with schizophrenia, supporting early intervention to reduce adverse outcomes.

## 1 Introduction

### 1.1 Overview of schizophrenia

Schizophrenia is a chronic, highly disabling disorder with a global lifetime prevalence of approximately 1% (1). In China, its prevalence is about 1.25%, which is significantly higher than that of other mental disorders (2). The overall prevalence in China is 0.28%, equivalent to approximately 4.05 million patients. Its onset is associated with genetic susceptibility, socioeconomic factors, education, and marital status (3). Patients with schizophrenia often require long-term medication and tend to lead sedentary lifestyles, which markedly increases their risk of physical comorbidities (4, 5). Approximately 37.5% of patients present with metabolic syndrome, and 23.1% with cardiovascular diseases (6). These comorbidities significantly increase mortality (7). For example, the incidence of pneumonia in patients with schizophrenia is about 10.3%, significantly higher than in the general population (OR: 2.62, 95% CI: 1.10–6.23) (8).

### 1.2 Overview of sarcopenic obesity

Sarcopenia is a progressive skeletal muscle disorder characterized by generalized loss of muscle mass and strength. It is associated with increased risks of falls, fractures, physical disability, and death (9). Meanwhile, obesity has become a major public health concern, as it increases the risk of chronic diseases (e.g., type 2 diabetes, coronary heart disease, and certain cancers) and shortens life expectancy (10). Sarcopenic Obesity (SO), a clinical syndrome combining excess body fat with sarcopenia, has gained attention for its synergistic effects, which worsen health consequences (11).

### 1.3 Relationship between sarcopenic obesity and schizophrenia

Existing studies indicate that SO prevalence in patients over 50 years can reach 23.12% (12). Most patients require long-term antipsychotic medication use (such as olanzapine and risperidone). These drugs disrupt endocrine balance: olanzapine significantly increases visceral fat (average +12.3%) and reduces skeletal muscle mass (-8.1%) by inhibiting the AMPK pathway and promoting adipogenesis, alongside elevated leptin levels (+45%) and upregulation of muscle catabolism markers (e.g., myostatin), accelerating muscle loss (13). Risperidone, though less associated with weight gain, may more strongly affect muscle mass through mitochondrial dysfunction (14). Antipsychotics also contribute to metabolic syndrome by impairing insulin signaling pathways, increasing fat storage, and reducing muscle protein synthesis. Additionally, they increase appetite via central nervous system regulation, further exacerbating obesity.

### 1.4 Overview of sarcopenic obesity diagnosis

Currently, SO diagnosis requires a combined assessment with methods such as dual-energy X-ray absorptiometry (DXA) for body composition measurement, muscle strength testing, and physical performance evaluation. However, these techniques have limitations, including high equipment cost, radiation exposure, and complex operation. In patients with schizophrenia, the feasibility of traditional imaging examinations is further limited by cognitive impairment and low treatment adherence, leading to a high rate of underdiagnosis. Moreover, the conclusions derived from different obesity indicators vary significantly, and different regions have different definitions of obesity.

### 1.5 Advantages of ultrasonographic diagnosis

Ultrasound is a noninvasive, portable, and repeatable imaging tool with unique advantages in muscle assessment. High-frequency ultrasound not only measures muscle thickness and cross-sectional area but also quantitatively analyzes the degree of muscle fat infiltration through echo intensity, enabling simultaneous assessment of muscle quality and fat distribution (15). Unlike conventional imaging, ultrasound offers real-time dynamic imaging, no radiation risk, and bedside accessibility, making it suitable for long-term follow-up and intervention in patients with schizophrenia.

### 1.6 Feasibility of thenar muscle ultrasonographic indices for diagnosing sarcopenic obesity

Hand muscles are susceptible to the effects of sedentary behavior and reduced activity. The thenar muscles, being small and superficial, are sensitive indicators of early muscle loss. In some chronic wasting diseases, hand function may be preserved longer than proximal upper limb function and remains essential for daily activities of daily living (16). Current muscle strength assessments often use a dynamometer, which relies on the thenar muscle contraction to enhance the grip strength index. Previous studies have confirmed a strong correlation between thenar muscle thickness and grip strength (17). Ultrasound indices related to thenar muscles are also associated with body fat content (18). Compared to other muscle groups (such as the quadriceps and gastrocnemius), thenar muscles are superficial and easily accessible, offering advantages in terms of portability and operational convenience. Multiple studies have confirmed the feasibility of using ultrasound to assess muscle thickness, cross-sectional area, and echo intensity for the diagnosis of sarcopenia (19, 20). Therefore, we explored the value of ultrasound in the diagnosis of SO. This study focused on the use of color Doppler ultrasound to measure thenar muscle thickness and echo intensity in patients with stable schizophrenia to evaluate their diagnostic value for SO. This aim was to provide an efficient and cost-effective solution for early SO screening and to develop new strategies for managing metabolic comorbidities in patients with schizophrenia.

## 2 Materials and methods

### 2.1 Study subjects

This was a cross-sectional study conducted from May to June 2024 at the Affiliated Zigong Hospital of Southwest Medical University. The study design is described using the PECO framework as follows: P (Population): Patients with stable schizophrenia (*n* = 490) meeting ICD-10 diagnostic criteria.

E (Exposure): Sarcopenic obesity (SO), diagnosed based on the AWGS 2019 criteria for sarcopenia and the Japanese consensus for obesity. C (Control): Patients without SO (*n* = 396). O (Outcome): Diagnostic performance of thenar muscle ultrasound for SO, and the association of SO with adverse outcomes (pneumonia and falls).Inclusion criteria were as follows: 1) Diagnosis of schizophrenia confirmed by a physician with at least an associate senior title in the department. 2. Normal cognitive function sufficient to complete the relevant examinations. 3. No significant symptom fluctuations or relapse within the past 6 months. 4. Regular use of medication at stable dosages. Exclusion criteria included: the presence of other severe mental illnesses or substance dependence, comorbid severe cardiovascular/cerebrovascular disease, advanced malignant tumors, a history of major trauma or surgery in the recent past, or neurological diseases affecting muscle structure or function.

### 2.2 Research methods

#### 2.2.1 Collection of basic data

A total of 505 patients were initially enrolled. After excluding 15 patients with incomplete data, all remaining 490 participants underwent both the index test (bilateral thenar muscle ultrasonography) and the reference standard (comprehensive diagnosis for SO), ensuring complete data for analysis. Demographic characteristics (sex, age, height, weight, smoking history, alcohol history, marital/childbearing history, and educational level), and the types and dosages of antipsychotic medications were recorded. To facilitate comparison, all antipsychotic dosages were converted into olanzapine-equivalent doses using the Defined Daily Dose method) (21, 22).

#### 2.2.2 Color doppler ultrasound examination

A GE Logiq E9 color Doppler ultrasound system equipped with a 12 MHz linear array probe was used. Patients were examined in the supine position with the palm facing upward and the hand relaxed. The probe was placed perpendicularly to the thenar muscle. On static images, the maximum thickness of the thenar muscle was measured by the same experienced ultrasonographer using built-in calipers. Each indicator was measured twice, and the average value was recorded.

#### 2.2.3 Echo intensity

Quantitative analysis was conducted using the open-source image processing tool ImageJ version 1.8.0. The specific workflow was as follows. First, the image scale was calibrated. The target region of interest (ROI) was manually delineated on the muscle cross-sectional image using the polygon selection tool, carefully excluding artifacts caused by poor probe-skin contact. The average grey intensity of the ROI was calculated using the measurement tool in the analysis module. The measurement protocol was designed as follows: Each thenar muscle was measured twice, and the average was taken as the value for that session. The procedure was repeated after a two-week interval, and the mean value from both sessions was used as the final assessment for each side.

#### 2.2.4 Measurement reliability

To assess the reliability of the ultrasound measurements, intra-rater reliability was evaluated by calculating the intraclass correlation coefficient (ICC) based on a two-way mixed-effects model for absolute agreement. The ICC for right thenar muscle thickness was 0.986, and for right thenar echo intensity was 0.880, and The ICC for left thenar muscle thickness was 0.987, and for left thenar echo intensity was 0.886, indicating excellent reliability.

### 2.3 Comprehensive diagnosis

#### 2.3.1 Operational procedures

All patients underwent body composition analysis using an InBody Body Composition Analyzer to measure fat mass, muscle mass, and related indicators. Muscle strength was assessed using a dynamometer (EH101; Xiangshan Company, China). Before testing, the device was calibrated, and the grip span was adjusted to fit the subject’s palm. During the test, subjects stood upright with feet together, upper limbs relaxed and hanging at the sides, and performed maximal instantaneous grip with the dominant hand. The subjects were required to keep their elbows extended and the trunk stable throughout the test, avoiding compensatory movements. Strength testing was performed on both limbs, and three consecutive independent tests were performed on each side with an interval of at least 60 s between tests. The arithmetic mean of three valid data points was used as the grip strength value (kg). Physical performance was assessed using the 6-meter walk test. Specifically, the subjects were instructed to walk 6 m in a straight line at their usual walking speed under a natural gait. The time required to complete the distance was recorded using a timing device, and the average walking speed was calculated accordingly and expressed in meters per second (m/s). This test method effectively reflects a subject’s lower limb motor function during daily activities.

#### 2.3.2 Sarcopenia diagnostic criteria

Sarcopenia was diagnosed according to the Asian Working Group for Sarcopenia (AWGS) 2019 Consensus on Sarcopenia Diagnosis and Management (23). Diagnosis required criterion (a) plus criterion (b) and/or (c): a. Muscle Mass: Appendicular Skeletal Muscle Index (SMI): Male <7.0 kg/m², Female <5.7 kg/m²; b. Muscle Strength: Grip Strength: Male <28 kg, Female <18 kg; c. Physical Performance: 6m Walk Test speed <1 m/s.

#### 2.3.3 Obesity diagnostic criteria

According to the Japanese Consensus Statement on Sarcopenic Obesity, obesity was diagnosed when either of the following was met: (a) Body fat percentage (BFP): Male ≥20%, Female ≥30% (24). (b) Visceral fat area (VFA) ≥100 cm² (25).

#### 2.3.4 Sarcopenic obesity definition

SO was defined as meeting both the sarcopenia and the corresponding obesity criteria above. Patients were categorized into the SO group or the non-SO group.

#### 2.3.5 Adverse outcomes

We assessed the association between SO and two key adverse clinical outcomes relevant to this population: pneumonia (an indicator of severe infection and respiratory morbidity) and falls (an indicator of physical disability and injury) a. Pneumonia: All patients underwent chest CT or chest radiography upon admission. Imaging results obtained within three months after the ultrasound were reviewed. Findings consistent with pneumonia were recorded as positive; otherwise, negative.b. Falls: Information on falls within the past year was collected from patients, attending physicians, nurses, and ward mates. A positive history of falls was recorded as positive; otherwise, negative.

### 2.4 Statistical methods

All data were entered into EXCEL and analyzed using SPSS statistical software (version 29.0). Continuous variables (thickness, echo intensity) were expressed as mean ± standard deviation (Mean ± SD); categorical variables (e.g., gender, marital status, age >60 years, pneumonia, falls) were expressed as frequency (percentage). Intergroup comparisons of continuous variables were performed using analysis of variance (ANOVA) and χ² tests for categorical variables. The association between adverse outcomes and SO was assessed using binary logistic regression. The relationship between ultrasound indices and adverse outcomes was also evaluated with binary logistic regression. Using comprehensive diagnosis as the gold standard, the diagnostic performance of ultrasound indices in diagnosing SO (sensitivity, specificity, and Youden index) was calculated. Receiver Operating Characteristic (ROC) curves were plotted, and the Area Under the Curve (AUC) was determined. Statistical significance was set at P < 0.05. Potential confounders considered in this study included age, sex, education level, smoking status, alcohol use, marital status, and antipsychotic dosage (olanzapine-equivalent). Effect modifiers such as sex and age were also evaluated through stratified analyses and interaction terms in regression models. To control for confounding, we performed both unadjusted and adjusted binary logistic regression analyses. In the adjusted models (Model 2), all aforementioned confounders were included to minimize their potential impact on the association between SO and adverse outcomes. To assess for potential effect modification, we introduced multiplicative interaction terms between SO and key variables (specifically sex, age [dichotomized at 60 years], and smoking history) into the fully adjusted models (Model 2) for each outcome. The significance of these interaction terms was evaluated using the likelihood ratio test. Since none of the interaction terms were statistically significant (all p-values > 0.05), we present only the results from the main effects model (Model 2) without stratification.”

## 3 Results

### 3.1 Comparison of general characteristics

Based on the comprehensive diagnosis, 94 out of 490 patients (19.2%) were identified with SO. As detailed in **Table 1**, the SO and non-SO groups were comparable in terms of age, alcohol history, and marital status (all *p* > 0.05). However, the SO group had a significantly higher proportion of males and smokers, and a lower proportion of individuals with education beyond the secondary level (all *p* < 0.01). Contrary to conventional expectation, the mean olanzapine-equivalent dose was significantly lower in the SO group compared to the non-SO group (*p* < 0.05).

**Table 1:** Demographic data.

Characteristic	SO (n=94)	Non-SO (n=396)	X²/t	P
Age (>60 years)	19 (20.2)	50 (12.6)	3.614	0.057
Education (>Secondary)	43 (45.7)	243 (61.4)	7.627	0.006
Smoking History (Yes)	55 (58.5)	171 (43.2)	7.183	0.007
Alcohol History (Yes)	25 (26.6)	87 (22.0)	0.922	0.337
Marital Status (Married)	17 (18.1)	93 (23.5)	1.272	0.259
Gender (Male)	78 (83.0)	252 (63.6)	12.924	0.001
Olanzapine Eq. Dose (mg)	11.52	13.92	2.099	0.036

### 3.2 Comparison of thenar muscle ultrasound indices both groups

Intergroup comparisons revealed a consistent and significant gradient in thenar muscle thickness across all measurement sites (left, right, dominant, non-dominant): SO group < Sarcopenic group < Control group (all p < 0.001). This indicates that the loss of muscle quantity is most severe in SO patients, followed by those with sarcopenia alone. However, a different pattern was observed for echo intensity, a marker of muscle quality. The SO group exhibited significantly higher echo intensity than the other two groups across all sites (all p < 0.01), as expected. A key finding, however, was that the echo intensity of the Control group was systematically higher than that of the Sarcopenic group across all measurement sites (e.g., dominant hand: 34.56 vs. 33.09; non-dominant hand: 36.01 vs. 33.70). This indicates that alterations in muscle quality do not parallel changes in muscle quantity. (**Table 2**).

**Table 2:** Differences in thenar muscle thickness and echo intensity between groups.

Indicator	SO (Mean ± SD)	Sarcopenic (Mean ± SD)	Control (Mean ± SD)	P
Thickness (mm)				
Left Thenar Muscle	13.81	13.76	14.87	<0.001
Right Thenar Muscle	14.82	14.97	16.07	<0.001
muscle of the dominant hand	14.77	14.88	16.00	<0.001
muscle of the non-dominant hand	13.87	13.85	14.93	<0.001
Echo Intensity				
muscle of the dominant hand	38.63	33.09	34.56	0.006
muscle of the non-dominant hand	39.45	33.70	36.01	0.004
Left Thenar Muscle	39.57	34.37	35.81	0.003
Right Thenar Muscle	38.52	32.42	34.75	0.006

### 3.3 Diagnostic performance of individual ultrasound indicators

Using comprehensive diagnosis as the gold standard, the AUC values for individual ultrasound indicators (left/right/dominant hand thickness and left/right/dominant echo intensity/) in diagnosing SO ranged from 0.593 to 0.672, indicating acceptable diagnostic performance (**Table 3**).

**Table 3:** Diagnostic efficacy of thenar muscle indices for SO.

Variable	AUC	Sensitivity	Specificity
Left Thenar Muscle Thickness	0.672	0.840	0.460
Right Thenar Muscle Thickness	0.661	0.681	0.606
thenar muscle thickness of the dominant hand	0.656	0.588	0.681
thenar muscle thickness of the non-dominant hand	0.668	0.470	0.819
Left Thenar Muscle Echo Intensity	0.603	0.947	0.230
Right Thenar Muscle Echo Intensity	0.617	0.830	0.439
thenar muscle echo Intensity of the dominant hand	0.627	0.830	0.444
thenar muscle echo Intensity of the non-dominant hand	0.593	0.926	0.253

### 3.4 Diagnostic performance of combined indicators

**Figure 1:**
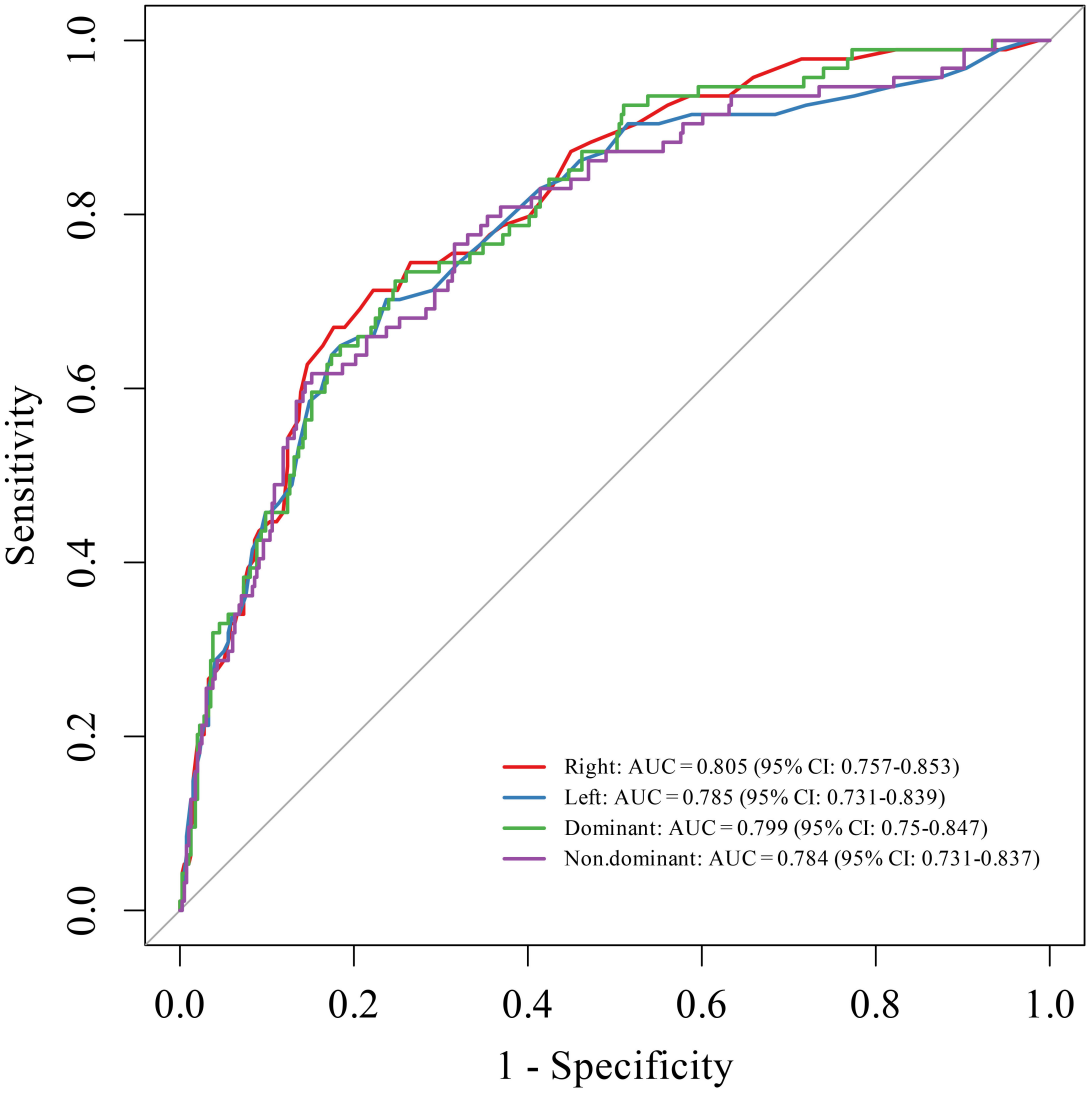
ROC curves for the combined diagnosis of SO.

Using comprehensive diagnosis as the gold standard, The combination of right thenar muscle thickness, right thenar muscle echo intensity, and gender yielded AUC, sensitivity, and specificity values of 0.805, 0.732, and 0.763, respectively, demonstrating a higher diagnostic efficacy (**Figure 1**, **Table 4**). Based on this, a Logistic regression diagnostic equation was constructed using right thenar Muscle Thickness, right Echo Intensity, and gender: logit (P) = -0.655 × muscle thickness + 0.043 × echo intensity + 2.632 × sex (male =0, female =1) + 5.141. The optimal cutoff value was 0.215.

**Table 4:** Diagnostic efficacy of combined models.

Variable	AUC	Sensitivity	Specificity
Left Combined with Gender	0.785	0.649	0.823
Right Combined with Gender	0.805	0.732	0.763
Dominant Combined with Gender	0.799	0.723	0.753
Non-dominant Combined with Gender	0.784	0.466	0.848

Left combined with gender: left thenar muscle thickness combined with left echo intensity and gender; Right combined with gender: right thenar muscle thickness combined with right echo intensity and gender. Dominant combined with gender: dominant hand thenar muscle thickness combined with dominant hand echo intensity and gender. Non-dominant combined with gender: non-dominant hand thenar muscle thickness combined with non-dominant hand echo intensity and gender.

### 3.5 Association between SO and adverse outcomes

We first compared the baseline characteristics between patients with and without each adverse outcome (**Tables 5**, **6**). Compared with the normal group, the SO group had an increased risk of both pneumonia and falls (**Table 7**, Model 1). After adjusting for potential confounding factors, the risk of falls remained significantly elevated (**Table 7**, Model 2). The association between SO and adverse outcomes is presented from the fully adjusted model (Model 2). Tests for effect modification by sex, age, and smoking history did not reveal any significant interactions (all p > 0.05); therefore, stratified analyses were not performed.

**Table 5:** Differences between fall and non-fall groups.

Characteristic	Fall (n=50)	No Fall (n=440)	X²/t	P
Age (>60 years)	13 (26.0)	56 (12.7)	6.537	0.011
Education (>Secondary)	29 (58.0)	257 (58.4)	0.003	0.956
Smoking History (Yes)	27 (54.0)	199 (45.2)	1.391	0.238
Alcohol History (Yes)	8 (16.0)	104 (23.6)	0.922	0.337
Marital Status (Married)	9 (18.0)	101 (23.0)	0.633	0.426
Gender (Male)	30 (60.0)	300 (68.2)	1.367	0.242
Sarcopenic Obesity (Yes)	18 (36.0)	76 (17.3)	10.157	0.001

We found that between the fall and non-fall groups, differences were observed only in age and sarcopenic obesity status; no differences were found in education level, smoking history, alcohol history, marital status, or sex.

**Table 6:** Differences between pneumonia and non-pneumonia groups.

Characteristic	Pneumonia (n=36)	No Pneumonia (n=412)	X²/t	P
Age (>60 years)	9 (25.0)	56 (13.6)	3.473	0.062
Education (>Secondary)	16 (44.4)	245 (59.5)	0.080	3.072
Smoking History (Yes)	8 (22.2)	99 (24.0)	0.807	0.059
Alcohol History (Yes)	8 (16.0)	104 (23.6)	0.922	0.337
Marital Status (Married)	9 (25.0)	89 (21.6)	0.636	0.224
Gender (Male)	26 (72.2)	282 (68.4)	0.220	0.639
Sarcopenic Obesity (Yes)	12 (33.3)	24 (6.70)	4.460	0.035

We found that between the pneumonia and non-pneumonia groups, a difference existed only in sarcopenic obesity status; there were no significant differences in other characteristics.

**Table 7:** Correlation between sarcopenic obesity and adverse outcomes.

Variable	Model1		Model2	
	P	OR	P	OR
Normal	–	1	–	1
Pneumonia	0.038	2.175	0.143	1.784
Fall	0.002	2.694	0.002	2.889

Model 2 was adjusted for potential confounders (e.g., age, sex, education, and smoking).

## 4 Discussion

### 4.1 High diagnostic efficacy of the thenar muscle ultrasound combined with gender model

To our knowledge, this is the first study to investigate the value of ultrasonographic assessment of thenar muscle indices in diagnosing SO among patients with schizophrenia. The results indicate that using thenar muscle thickness or echo intensity alone provides acceptable diagnostic efficacy (AUC 0.593–0.672), with high sensitivity but limited specificity. However, when gender was incorporated, diagnostic efficacy significantly improved (right combined AUC = 0.805), with both sensitivity and specificity reaching desirable levels (0.732, 0.763). This finding is consistent with previous studies that used ultrasound measurements of other muscle sites (thickness and echo intensity) for sarcopenia (21) diagnosis. The Logistic regression model developed in this study, based on right thenar muscle thickness, echo intensity, and sex, offers a novel, noninvasive, and convenient tool for screening SO in patients with schizophrenia.

### 4.2 A phenotypic distinction: dissociation of muscle mass and quality in sarcopenia and sarcopenic obesity

“Our finding of reduced muscle thickness coupled with relatively low echo intensity in the sarcopenia-alone group suggests a muscle alteration pattern dominated by a loss of contractile tissue with comparatively less fat infiltration (**Table 2**). This profile aligns with descriptions of sarcopenia subtypes primarily driven by factors such as age-related anabolic resistance or inadequate nutrition (9), rather than the profound lipid overflow and metabolic dysfunction characteristic of obesity. This underscores the well-recognized heterogeneity in the pathophysiology of sarcopenia (26, 27).

### 4.3 High prevalence of SO in patients with stable schizophrenia

Among patients with stable schizophrenia in this study, the prevalence of SO was 19.2%, which is substantially higher than that reported in the general population (28, 29). This elevated prevalence may be closely related to long-term use of antipsychotic medications and unhealthy lifestyle factors. Second-generation antipsychotics (e.g. olanzapine and clozapine) commonly induce insulin resistance, dyslipidemia, and weight gain, while also inhibiting muscle protein synthesis and promoting fat accumulation (4, 30). The proportions of males (83.0%) and smokers (58.5%) were significantly higher in the SO group than in the non-SO group. Male patients may be more susceptible to muscle loss due to declining testosterone levels and differences in muscle metabolism, consistent with findings by Tanioka et al. regarding sex-related sarcopenia risk in schizophrenia (supported by literature on the role of testosterone) (5). Sedentary behavior and smoking may further exacerbate muscle catabolism and fat infiltration by promoting oxidative stress and chronic low-grade inflammation (e.g., activation of TNF-α/NF-κB pathways) (12). The proportion of individuals with higher education (> secondary) was lower in the SO group (45.7% vs. 61.4%), which aligns with previous studies (31, 32). Higher education is generally associated with better health literacy, stronger disease prevention awareness, and healthier lifestyle management (e.g., balanced diet, regular exercise, and avoidance of risky behaviors), as well as improved medication adherence, thereby reducing the risk of SO risk (33, 34). This study found no significant differences between the SO and non-SO groups in terms of age, alcohol history, or marital status, which may reflect differences in sample selection or information bias due to patient cognitive impairment. An interesting finding was that the antipsychotic drug (olanzapine-equivalent) dose in the SO group was lower than in the non-SO group (11.52 mg vs 13.92 mg). This contradicts the conventional view that antipsychotic drugs are risk factors for obesity (35). Possible explanations include the fact that patients receiving higher doses may have better symptom control, enabling them to accept and participate in health management (e.g., nutritional support and exercise interventions), thereby reducing SO risk. Notably, all doses in this study were below the reported optimal olanzapine dose of 15.2 mg/d. Furthermore, the “obesity paradox” phenomenon–where obesity may exert a protective effect in certain chronic diseases–has also been reported in cardiovascular disease and sarcopenia (36–38). However, the complex relationship among antipsychotic drugs, obesity, and sarcopenia requires further investigation.

### 4.4 SO is closely associated with adverse outcomes

This study found that SO significantly increased the risk of falls (adjusted odds ratio [OR], 2.9). Decreased muscle mass and function directly impair balance and gait stability, reduce walking confidence, and increase fear of falling (39), often leading to a vicious cycle of “fear of falling → reduced activity → worsening SO” in patients with schizophrenia patients (40). SO combines the dual risk factors of sarcopenia (impaired balance) and obesity (altered center of gravity and restricted mobility), further exacerbating fall risk (41). Additionally, SO was associated with an increased risk of pneumonia (unadjusted OR≈2.2), with a particularly strong association in female patients (adjusted OR≈8.1). Potential mechanisms include: (a) Respiratory muscle dysfunction: Reduced mass and strength of respiratory muscles (diaphragm, intercostals) weaken lung function and cough efficacy, increasing the risk of aspiration pneumonia (42, 43). (b) Impaired immune function: Muscle tissue plays a key role in immune regulation. Sarcopenia weakens immune responses, reducing infection resistance (44). (c) Limited mobility: Reduced activity may decrease the ability to clear lung secretions, further increasing pneumonia risk (44). (d) Mechanical ventilation impairment: Fat accumulation in the chest wall and abdomen restricts lung expansion, reducing ventilation and increasing pneumonia risk (45). (e) Chronic inflammatory state: Chronic low-grade inflammation in obesity may further weaken immune function (46). (f) Comorbidity burden: Obesity is often accompanied by diabetes and cardiovascular diseases, which increase infection risk (47). The regression coefficients in this study were higher than those reported by Chou and F. Petermann-Rocha (48, 49). A possible reason is that their study populations included either patients with schizophrenia or SO, whereas the present study examined individuals with both conditions simultaneously. This highlights the interaction between SO and schizophrenia and supports the pathophysiological model linking sarcopenia with mortality proposed by Nimptsch et al. (10). This further emphasizes that clinicians must pay attention to both the patients’ mental health and the risk of SO. Importantly, after adjusting for confounding factors, SO remained significantly associated only with falls; its association with pneumonia was no longer statistically significant. This suggests that the observed correlation between SO and pneumonia was largely driven by confounders, particularly age-related pathophysiological changes. Moreover, the relatively short duration of this prospective study and the limited number of positive cases may have contributed to these findings. Therefore, these findings warrant cautious interpretation and should be validated in future studies with larger sample sizes and extended follow-up durations.

### 4.5 Strengths and limitations

Overall, this study provides a safe, objective, and reliable indicator of SO in patients with schizophrenia. Its strengths include: (a) Practicality: Ultrasound is simple, quick, and can be performed at the bedside or in outpatient settings without special patient preparation. Studies have reported severe cognitive impairment in 89.3% of patients with schizophrenia, making it difficult for these patients to cooperate autonomously with CT, prolonged MRI, or follow-up. Our method is easier to apply and promotes the use of this special patient population. (b) Advantages over traditional methods: Compared with traditional diagnostic methods, ultrasound is radiation-free and relatively inexpensive, reducing patient risk and financial burden. (c) Real-time assessment: Ultrasonography allows real-time dynamic observation of muscle morphology, structure, and blood perfusion, enabling the assessment of muscle mass and, to some extent, functional status. (d) Clinical relevance: The study explored the association between SO and key adverse outcomes (pneumonia and falls), underscoring the importance of early SO interventions.

However, this study also had some limitations. (a) Operator dependence: Ultrasound results may vary depending on operator experience, measurement site, and method. (b) Single-center design: A single-center study with a relatively limited sample size may have affected the generalizability of the results. (c) Standardization needed: Measurements were based on the physician’s experience. Standardization of ROI selection and grayscale analysis processes is needed, potentially incorporating AI algorithms (e.g., deep learning) to reduce human error. (d) Recall bias for falls: Assessment of falls relies on patient, doctor, and nurse recall, potentially missing positive cases due to memory bias. (e) Potential confounders: Although adjustments were made, unknown confounders may exist. Additional potential influencing factors (e.g., specific exercise levels and dietary details) should also be included. (f)Our study employed inconsistent definitions of pneumonia, and a discrepancy in specificity was noted between chest DR and CT. Future studies should aim to adopt unified diagnostic protocols.(g) Lack of Longitudinal Data: This was a cross-sectional study. Longitudinal studies are needed to validate the predictive value of ultrasound indices for adverse outcomes using more objective outcome assessments.

Therefore, further standardization of color Doppler ultrasound examination procedures, expansion of the sample size, and multicenter clinical studies are required. These efforts should be combined with additional muscle function assessment metrics to enhance the accuracy and reliability of ultrasound for diagnosing intramyopathic obesity. This will provide a more robust foundation for early diagnosis, treatment, and prognostic evaluation of this disease. There is also a need to apply more objective and rigorous methods to assess adverse outcomes and conduct longitudinal studies to validate the predictive value of ultrasound indicators. Furthermore, despite adjusting for confounding factors, unknown confounders may still exist. It is necessary to include additional potential influencing factors (such as specific physical activity levels and dietary details).

## 5 Conclusion

In conclusion, this study demonstrated that a diagnostic model combining color Doppler ultrasound measurements of thenar muscle thickness and echo intensity, combined with sex, achieves high diagnostic efficacy (AUC > 0.8) for screening SO in patients with stable schizophrenia. Its noninvasive, economical, and portable advantages make it a practical tool for the early identification of high-risk patients in clinical settings. For SO risk factors (e.g., male sex, smoking, and sedentary behavior), interdisciplinary management strategies are urgently needed, incorporating metabolic monitoring, lifestyle interventions (nutrition, exercise), and rational antipsychotic medication adjustments. Such strategies are essential to reduce adverse outcomes–such as pneumonia and falls–and ultimately improve the long-term quality of life and prognosis of patients with schizophrenia.

## Data Availability

The raw data supporting the conclusions of this article will be made available by the authors, without undue reservation.

## Glossary

Sarcopenic Obesity: A condition characterized by the coexistence of reduced skeletal muscle mass and strength (sarcopenia) with excess body fat (obesity), which increases risk of disability, metabolic disorders, and poor health outcomes
Schizophrenia: A chronic psychiatric disorder marked by disturbances in thought processes, perceptions, emotional responsiveness, and social interactions. Antipsychotic medications and lifestyle factors can predispose patients to metabolic complications
Thenar Muscles: A group of small muscles located at the base of the thumb (in the palm), responsible for thumb movement and grip strength
Ultrasonography: A medical imaging technique that uses high-frequency sound waves to visualize internal structures, such as muscles, organs, or vessels, and is valued for being non-invasive, safe, and relatively low-cost
